# Anal Human Papillomavirus Genotyping among HIV-Positive Men Who Have Sex with Men in Xi’an, China

**DOI:** 10.1371/journal.pone.0125120

**Published:** 2015-04-29

**Authors:** Zhen Li, Haoran Zhang, Xiangwei Li, Yu Yang, Henan Xin, Mufei Li, Boxuan Feng, Lei Gao

**Affiliations:** 1 Institute of Pathogen Biology, Chinese Academy of Medical Sciences & Peking Union Medical College, Dong Dan San Tiao No. 9, Beijing, 100730, China; 2 Department of Epidemiology and Biostatistics, School of Public Health, Peking University Health Science Centre, Beijing, 100191, China; National Center for AIDS/STD Control and Prevention, CHINA

## Abstract

**Background:**

Anal human papillomavirus (HPV) infection and its related diseases are relatively common in men who have sex with men (MSM), especially in those HIV positive. In China, molecular epidemiology of anal HPV infection among HIV-positive MSM has been sparsely studied.

**Methods:**

A cross-sectional study was conducted among HIV-positive MSM in Xi’an, China between April and July 2014. Anal swabs were collected for HPV genotyping.

**Results:**

A total of 195 HIV-positive MSM were included in this study. HPV genotyping showed that 99.0% (191/193) of participants were positive for at least one of the targeted 37 HPV genotypes. 183 (94.8%) of them were infected with multiple high-risk types and 154 (79.8%) of them with low-risk HPV types. HPV 18 was the most frequently identified high-risk type, followed by HPV 16 and HPV 51. As for low-risk types, HPV11, HPV 6 and HPV 81 were most commonly observe. High-risk HPV infection was found to be associated with the status of antiretroviral therapy (ART), the distribution of low-risk types was observed to be varied by CD4^+^ T cell level.

**Conclusion:**

Almost all HIV-positive MSM were anal HPV infected in our study. It is highly recommended to consider regular active screening and preventive intervention of HPV infection among this high risk population.

## Introduction

Human papillomavirus (HPV) infection is one of the most common sexually transmitted infections worldwide, representing a significant health problem due to its high prevalence and transmissibility [1]. The human immunodeficiency virus (HIV) infection has been suggested to make humans more susceptible to HPV infection because of the attenuated immune system [2,3]. It has been widely accepted that men have sex with men (MSM) is a high-risk population for both HPV and HIV infection. A recent systematic review and meta-analysis included 53 studies reported that the prevalence of the HPV co-infection was 89–93% in HIV infected MSM [4]. Additionally, anal HPV infection is one of the main causes of anal cancer, and the incidence of anal cancer is substantially higher in MSM than general population, especially in HIV positives [5–8]. It is worth to notice that the prevalence of HIV infection in MSM is increasing in China in recent years. Until 2013, it was estimated that nearly 63,730 MSM were living with HIV infection in China. The control of HPV infection and its related diseases is very important for improving the living quality of HIV-positive MSM. However, anal HPV infection and genotype distribution in the HIV-positive MSM has not been widely studied in China.

Our previously studies reported around 60% HIV negatives and 90% HIV positives were anal HPV infected in MSM from China [9,10]. To improve our understanding of anal HPV infection and its related pre-cancerous diseases, we conducted a pilot study among 95 HIV-positive MSM in Beijing, and the prevalence of abnormal anal cytology was found to be 37.9% [11]. Based on such previous work, we expanded the sample size of HIV-positive MSM in Xi’an city to explore the prevalence and distribution of anal HPV genotypes. Xi’an is the capital city of Shaanxi province, more than 1700 HIV infections had been reported in Xi’an until the end of 2012. Between 2007 and 2012, the proportion of homosexual transmission in the total HIV infections was increased from 14.3% to 56.7%, which had become the major route of transmission in Xi’an [12].

## Materials and Methods

### Ethic statement

The study was approved by the Ethics Committees of the Institute of Pathogen Biology, Chinese Academy of Medical Sciences & Peking Union Medical College. Written informed consent was obtained from each study participant before the interview and testing.

### Study population

The study was conducted in the Eighth People’s Hospital in Xi’an from April to July 2014. Study participants were recruited through a local nongovernment organization (Xi’an Tongkang Volunteers Workstation). Multiple methods were used for recruitment including website advertisements, distributing flyers with study-related information at MSM frequented venues (e.g., MSM clubs, bars, parks and bathhouses), and eligible study participants were also encouraged to refer their peers to attend the study. Those eligible participants were HIV-seropositive males, at least 18 years old, ever had homosexual behaviors, willing to provide anal swabs and blood for the test, and physically able and willing to provide written informed consent. Study participants who were tested HPV positive were informed by study personnel confidentially and referred to treatment at the Institute of STD/AIDS Prevention and Treatment, Xi’an District Center for Disease Prevention and Control and the STD/AIDS clinic of Xi’an eighth hospital.

### Data collection

Self-reported socio-demographic characteristics (e.g., age, income, education, employment, and marriage status), antiretroviral therapy (ART) status and sexual behaviors in the past 6 months data were collected through one-to-one interviews by the trained interviewer in a separate room using a standardized questionnaire. Each study participant was assigned a unique code that was used to link the questionnaire and specimens. Personal contact information, which was blinded to researchers, was kept by the Xi’an Tongkang Volunteers Workstation for test results feedback and data validation. CD4^+^ T cell counts and HPV genotypes were collected for blood test and anal swabs test, respectively.

### Sample collection and laboratory tests

Blood samples were collected for testing CD4^+^ T cell counts (BD FACSCountsystem). Trained personnel collected anal samples by rotating a saline water moistened nylon flocked swab in the anal canal for about 2 minutes. The swab was then kept in 3 mL of sample transport medium for Hybribio HPV DNA Test. Hybribio HPV DNA Test is based on flow-through hybridization to identify HPV types. The denatured DNA was placed into sample wells containing specific probes on the Hybrimem HPV-37 membrane to determine HPV types. The final results were detected by colorimetric change on the membrane under direct visualization. Positive and negative controls were included in the GenoArray test kit in every PCR analysis as well as during the hybridization process for quality. Mixtures of different specific probes can be used in the same well of a 42-well plate format allowing for multiplex analysis. HPV were classified as low-risk (LR) type and high-risk (HR) type according to the kit instruction [11,13]. Hybribio HPV DNA Test provides identification of 21 recommended HR subtypes (16, 18, 26, 31, 33, 34, 35, 39, 45, 51, 52, 53, 55, 56, 58, 59, 66, 68, 69, 82, and 83) which are associated with cervical cancer. Besides that, the kit also provides detection of 16 LR subtypes (6, 11, 40, 42, 43, 44, 54, 57, 61, 67, 70, 71, 72, 73, 81, and 84).

### Statistical analysis

Questionnaires and laboratory results were double entered and compared with EpiData software (EpiData 3.02 for Windows, The Epi Data Association Odense, Denmark). After cleaning, the data was analyzed using SPSS (version 15.0 for Windows; SPSS Inc., Chicago, IL, USA). The characteristics of study population were showed by age, education, marriage status, self-reported sexual orientation, ever had sex with women, age at the first homosexual act, whether anal sex is a regular sex behavior or not. Based on CD4^+^ T cell level (divide by the median value of 394 cells/μL) or ART status (started or not), subjects were classified into two categories, respectively. Differences between HPV-types in these variables were assessed with Pearson chi-square test. Frequency distributions are presented for qualitative variables, medians, and inter-quartile ranges (IQRs) are presented for quantitative variables with asymmetrical distributions. The general description of the principal variables including central tendency and dispersion (mean, standard deviation, median, percentiles) for the quantitative variables was performed. To identify potential variables related with HPV infection, univariate analysis were performed using Pearson’s chi-square test. All variables with p-values < 0.05 in univariate analysis were entered into the unconditional multiple logistic regression analyses, and the associations were then assessed by means of odds ratios (OR) and 95% confidence intervals. Age, ART status and CD4 level were fixed in the multiple logistic regression models.

## Results

### Participant Characteristics

A total of 195 eligible HIV-positive MSM were enrolled. HPV genotyping results and current CD4^+^ T cell counts were available for 193 (99.0%) and 170 (87.2%), respectively. The major characteristics of the study participants are listed in Table 1. Overall, the mean (standard deviation, SD) age was 34±9 years with a range of 18–60 years. More than a third of them (38.5%) were 20–29 years old. Around half of the participants were single (52.3%) and 64.1% of them have received education more than 12 years. The median current CD4^+^ T cell count was 394 cells/μL (range, 259–517 cells/μL). A vast majority (86.2%) of them ever had started ART.

**Table 1:** Characteristics of the study population.

Variables	n (%)
**Total**	195
**Age**	
Mean ± SD (range)	34±9 (18–60)
< 20 years	3 (1.5)
20–29 years	75 (38.5)
30–39 years	61 (31.3)
≥ 40 years	56 (28.7)
**Education level**	
≤ 9 years	25 (12.8)
9–12 years	45 (23.1)
> 12 years	125 (64.1)
**Current marital status**	
Single	102 (52.3)
In marriage	65 (33.3)
Divorced/widowed	28 (14.4)
**Antiretroviral treatment (ART)**	
Started	168 (86.2)
Not started	27 (13.8)
**Median CD4** ^**+**^ **T cell counts (IQR)#**(cells/μL)	394 (259, 517)

#23 subjects’ blood samples were unavailable for testing CD4+T cell counts.

### Anal HPV-types Distribution

As shown in Table 2, the genotyping results of HPV were available for 193 participants. 99.0% (191/193) of them were positive for at least one of the targeted 37 HPV types. 94.8% (183/193) of them were multiple HR-types infected, and 79.8% (154/193) were LR-HPV infected.

**Table 2:** Association between characteristics and HPV types distribution in HIV-positive MSM.

	Any HPV	High-risk HPV	Low-risk HPV
	n(%)	n(%)	n(%)
**Total** ^**#**^ **(N = 193)**	191 (99.0)	183 (94.8)	154 (79.8)
**Age**			
< 20 years	3/3 (100.0)	3/3 (100.0)	1/3 (33.3)
20–29 years	72/74 (97.3)	69/74 (93.2)	57/74 (77.0)
30–39 years	60/60 (100)	58/60 (96.7)	49/60 (81.7)
≥ 40 years	56/56 (100)	53/56 (94.6)	47/56 (83.9)
***p* value**	0.355	0.712	0.186
**Education level**			
≤ 9 years	25/25 (100.0)	25/25 (100.0)	22/25 (88.0)
9–12 years	44/45 (97.8)	42/45 (93.3)	39/45 (86.7)
> 12 years	122/123 (99.2)	116/123 (94.3)	93/123 (75.6)
***p* value**	0.595	0.570	0.185
**Current marital status**			
Single	99/101 (98.0)	96/101 (95.0)	77/101(76.2)
In marriage	65/65 (100.0)	62/65 (95.4)	55/65 (84.6)
Divorced/widowed	27/27 (100.0)	25/27 (92.6)	22/27 (81.5)
***p* value**	0.646	0.811	0.409
**Antiretroviral treatment (ART)**			
Started	166/166 (100.0)	160/166 (96.4)	134/166 (80.7)
Not started	25/27 (92.6)	23/27 (85.2)	20/27 (74.1)
***p* value**	**0.019**	**0.036**	0.286
**CD4** ^**+**^ **T cell level** *			
< 394 cells/μL	84/85(98.8)	80/85(94.1)	73/85(85.9)
≥ 394 cells/μL	84/85(98.8)	82/85(96.5)	62/85(72.9)
***p* value**	1.000	0.720	**0.037**

^#^ 2 anal samples were unavailable for HPV genotyping.

*23 subjects’ blood samples were unavailable for testing CD4+T cell counts.

**Fig 1:**
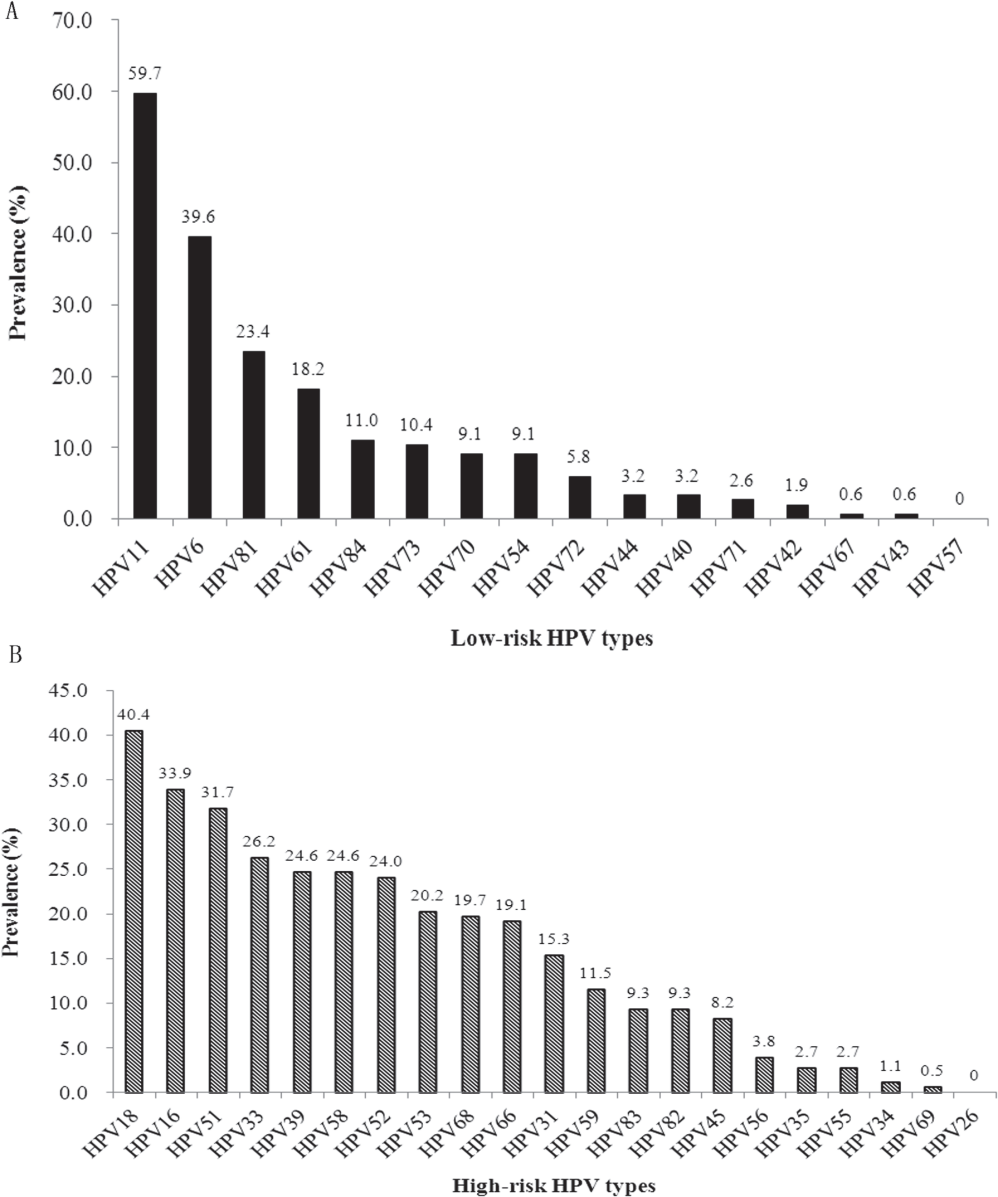
Prevalence of HPV types in 193 HIV-positive MSM. The numbers above the bars indicate the proportion of the patients in each category. HPV 11 and HPV 6 were found to be the most prevalent low-risk types, HPV 18 and HPV 16 were most common high-risk types among the study population.

Fig 1A showed the prevalence of type-specific HPV infection. HPV 11 (59.7%) and HPV 6 (39.6%) were found to be the most frequently identified LR-HPV. The following prevalent LR-HPV genotypes were: HPV 81 (23.4%), HPV 61 (18.2%), HPV 84 (11.0%), HPV 73 (10.4%), and HPV 70 (9.1%). As depicted in Fig 1B, among the HR-HPV types, HPV 18, HPV 16, and HPV 51 were most frequently identified (40.4%, 33.9% and 31.7%, respectively). HPV 33, along with HPV 39, HPV 58, HPV 52, HPV 53, HPV 68, HPV 66, HPV 31, and HPV 59 were the following common detected HR-HPV types, with prevalence of 26.2%, 24.6%, 24.6%, 24.0%, 20.2%, 19.7%, 19.1%, 15.3%, and 11.5%, respectively.

### HPV Distribution by CD4^+^ T cell Level or ART Status

As shown in Table 2 and Table 3, ART and condom using were significantly associated with any-type HPV infection (*p* = 0.019 and *p* = 0.044, respectively). ART status, role of sexual behaviors and un-preferred respective sexual behavior were associated with HR-HPV infection (*p* = 0.036, *p* = 0.004 and *p* = 0.031, respectively), CD4^+^ T cell level (*p* = 0.037) and having a fixed sexual partner (*p* = 0.019) were related with LR-HPV infection in univariate analysis. In multiple logistic regression analysis, as shown in Table 4, higher CD4^+^T cell level was found to be associated with significantly increased rate of LR-HPV infection (adjusted OR: 2.99, 95%CI: 1.26–7.08) and started ART was associated with decreased risk of HR-HPV infection (adjusted OR: 0.13, 95%CI: 0.02–0.71).

**Table 3:** Association between sexual behaviors and HPV types distribution in HIV-positive MSM.

	Any HPV	High-risk HPV	Low-risk HPV
	n (%)	n (%)	n (%)
**Total** ^**#**^ **(N = 193)**	191(99.0)	183(94.8)	154(79.8)
**Had sexual behaviors with women** †			
Yes	113/113(100)	107/113(94.7)	94/113(83.2)
No	78/80(97.5)	76/80(95.0)	60/80(75.0)
***p* value**	0.171	1.000	0.203
**Had used condom in heterosexual behaviors**			
Yes	28/28(100)	26/28(92.9)	23/28(82.1)
No	85/85(100)	81/85(95.3)	71/85(83.5)
***p* value**	**-**	0.636	1.000
**Having fixed sexual partner** †			
Yes	58/58(100)	56/58(96.6)	40/58(69.0)
No	133/135(98.5)	127/135(94.1)	114/135(84.4)
***p* value**	1.000	0.726	**0.019**
**Role of homosexual behaviors** *			
Only insertive	21/22(95.5)	19/22(86.4)	17/22(77.3)
Only receptive	38/39(97.4)	34/39(87.2)	31/39(79.5)
Insertive or receptive	127/127(100)	125/127(98.4)	102/127(80.3)
***p* value**	0.094	**0.004**	0.946
**Only insertive** *			
Yes	21/22(95.5)	19/22(86.4)	17/22(77.3)
No	169/170(99.4)	163/170(95.9)	136/170(78.0)
***p* value**	0.217	0.092	0.780
**Only receptive** *			
Yes	38/39(97.4)	34/39(87.2)	31/39(79.5)
No	152/153(99.3)	148/153(96.7)	122/153(79.7)
***p* value**	0.366	**0.031**	1.000
**Had used condom in the homosexual behaviors**			
Yes	89/89(100)	85/89(95.5)	67/89(75.3)
No	22/24(91.7)	22/24(91.7)	18/24(75.0)
***p* value**	**0.044**	0.606	1.000
**Had group sexual behaviors in the past 6 months**			
Yes	5/5(100)	5/5(100)	3/5(60.0)
No	186/188(98.9)	178/188(94.7)	151/188(80.3)
***p* value**	1.000	1.000	0.266

^#^23 subjects’ blood samples were unavailable for testing CD4+T cell counts.

*Casual sexual partner and sexual behaviors pattern results were missing for one sample.

†Fisher test.

**Table 4:** Factors correlated with HPV sub-types infection among Xi’an MSM by multivariate logistic regression.

	High-risk HPV		Low-risk HPV	
	*p*	Adjusted OR(95%CI)	*p*	Adjusted OR(95%CI)
**Total** ^**#**^ **(N = 193)**	183 (94.8%)		154(79.8)	
**Age**				
< 20 years		Ref.		Ref.
20–29 years	0.964	999.9 (<0.01->999.9)	0.584	0.47 (0.03–6.96)
30–39 years	0.964	999.9 (<0.01->999.9)	0.549	0.44 (0.03–6.61)
≥ 40 years	0.966	999.9 (<0.01->999.9)	0.637	0.52 (0.03–8.09)
**CD4+T cell level**				
< 394 cells/μL		Ref.		Ref.
≥ 394 cells/μL	0.121	0.27 (0.05–1.42)	0.013	2.99 (1.26–7.08)
**Antiretroviral treatment (ART)**				
Not started		Ref.		Ref.
Started	0.019	0.13 (0.02–0.71)	0.821	0.88 (0.27–2.79)
**Role of homosexual behaviors** *				
Only insertive		Ref.		
Only receptive	0.425	2.14 (0.33–14.03)		
Insertive or receptive	0.114	0.18 (0.02–1.52)		
**Having fixed sexual partner**				
Yes				Ref.
No			0.148	1.86 (0.80–4.28)

^#^ 2 anal samples were unavailable for HPV genotyping.

* 23 subjects’ blood samples were unavailable for testing CD4+T cell counts.

^Adjusted for age, education, and current marital status.

**Fig 2:**
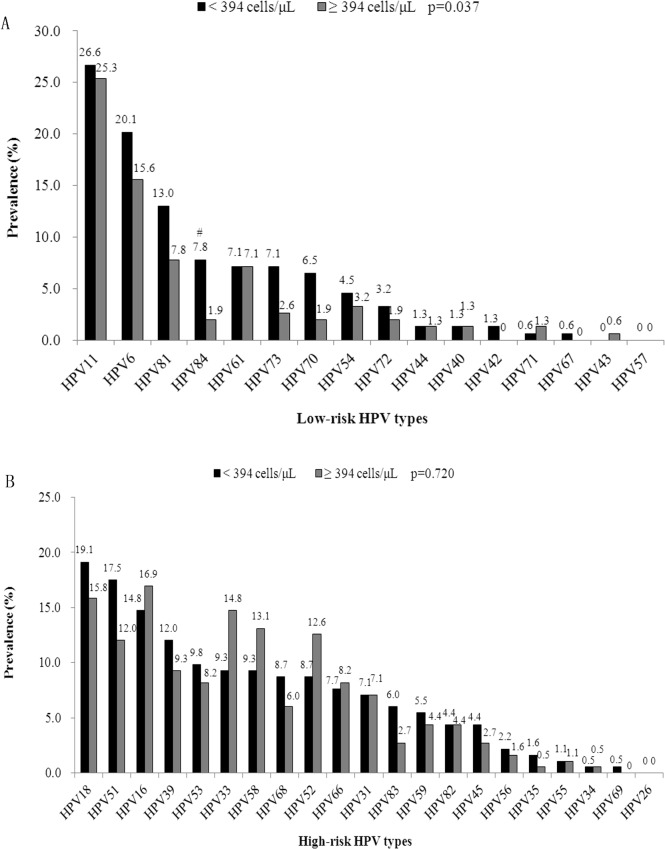
The prevalence of HPV types in 193 HIV-positive MSM by CD4 level. The study population was grouped by the median values of CD4^+^ T cell level (394 cells/μL). The numbers above the bars indicate the prevalence of each type. #HPV84 was significantly associated with CD4+T cell count level, the prevalence in “< 394 cells/μL” group was higher than that in “≥ 394 cells/μL” group (*p* = 0.015).

HPV types distribution stratified by median CD4^+^ T cell counts (Fig 2A) showed that distribution of HPV 84 was significantly associated with CD4^+^ T cell level (*p* = 0.015). As shown in the Fig 2B, for participants with lower level of CD4^+^ T cell, HPV 18, HPV 51, and HPV 16 were the most frequently identified HR-HPV types, but HPV 16, HPV 18, and HPV 33 were found to be the most frequently identified types among subjects with higher CD4^+^ T cell counts ≥ 394 cells/μL.

**Fig 3:**
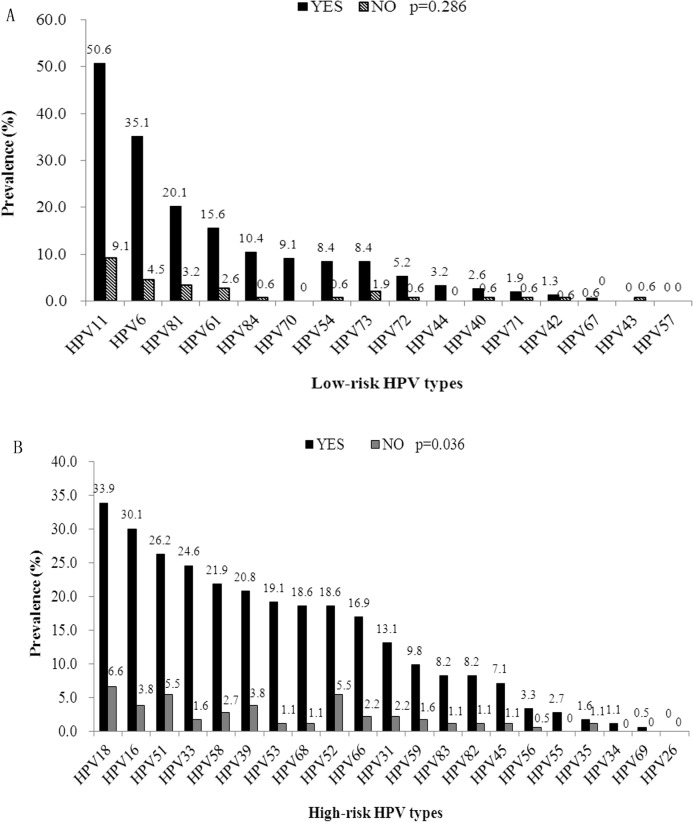
Comparison of prevalence of HPV types in 193 HIV-positive MSM by ART status. The numbers above the bars of low-risk HPV types indicate the proportion of each low-risk types in the 154 patients infected with low-risk HPV types. Likewise, the numbers above the bars of high-risk HPV types indicate the proportion of each high-risk type in the 183 patients infected with high-risk HPV types. The distribution of high-risk HPV types was associated with ART status (*p* = 0.036).

When stratified by ART status, HPV 11, HPV 6, and HPV 81 were the most frequently identified types among LR-HPV types regardless of ART status. As for HR-HPV types group, HPV 18, HPV 16, and HPV 51 were most frequently detected among those who had started ART. But for those who had not started ART, HPV 18, HPV 52, and HPV 51 were most frequently identified. No significant different distribution of LR-HPV types was found by ART status as shown in Fig 3.

## Discussion

The present study aims to investigate the HPV genotype distribution among HIV-infected MSM in China. Overall, we found a high prevalence of any-HPV (99.0%), HR-HPV (94.8%), and LR-HPV (79.8%) infection in our study participants. The prevalence of any-HPV in the present study was close to a meta-analysis (96.2%), Lei Gao et al.(96.0%) and Xj Zhang et al. (96.6%) reported [9,10,14], but was higher than other studies (65.5%-82.1%) [11,13,15] in China. Among HR-HPV types, HPV18 was the most common, followed by HPV 16, HPV 51. As for LR-HPV types, HPV 11, HPV 6 and HPV 81 were the most frequently detected. The distribution of HPV 11/6/18/16 were basically in line with other studies, but other HPV types distribution were different in different regions and studies [9–11,13,15]. As for the discordant correlations between anal HPV infection and CD4 count or ART in different researches [16–19], our findings showed that HIV-positive MSM with a CD4^+^ T cell level higher than 394 cells/μL may be more susceptive to infection by LR-HPV than those with less than that and ART might be a protective factor for HR-HPV infection.

Anal cancer is a relatively rare disease compared to cervical cancer. Its annual incidence is less than 2 in 100,000 persons in the general population worldwide, but is high among well-defined populations such as MSM, particularly HIV-infected MSM, in whom the incidence is up to 80-fold higher than that in the general population [20,21]. A large proportion of anal cancer is caused by anal infections with carcinogenic HPV [22], and among them, approximately 80% to 85% cases were caused by infection of HR-HPV such as HPV 16 or 18 [23,24]. Even HPV infection is quite common in HIV-positive population, HPV 16 is still the most frequently found HPV type related to anal intraepithelial neoplasia and anal cancer [25]. In the present investigation, a wide array of HPV types was identified and multiple infections were found to be common, it is consistent with the reports from previous studies [14,15,26]. In our study participants, HPV 18 and 16 were most frequently identified HR-HPV types. High prevalence of HPV 51 was also observed. Such HR-HPV types might increase the risk of HPV related anal cancer and might be targeted for the screening HPV infection and monitoring pre-cancer in HIV-positive MSM. But further analyses are necessary to confirm such distribution is common in HIV infected MSM in China.

The presence of LR-HPVs, in particular HPV 6 and HPV 11 has been reported for HPV-related invasive anal carcinoma as well [27,28]. The prevalence of HPV 6 and/or HPV 11 in the anal canal of MSM has been shown to be 15% in HIV negatives and as high as 60% in HIV positives [29,30]. In our present study, HPV 11 and HPV 6 were also the most common LR-HPV types in HIV infected MSM. The HPV vaccine covering HPV-6, 11, 16 and 18 subtypes has been shown to be effective in preventing anogenital warts and anal intraepithelial neoplasia in men [31–34]. While the biggest challenge for vaccination will be to identify and vaccinate before HPV acquisition, thus vaccination before sexual activity commences is the optimal strategy. There are data supporting the alternative approach of vaccinating young MSM after sexual debut [35–37]. However, the actual situation is that HPV vaccination among males has not been suggested and widely studied in China. The introduction of a routine preventive screening and treatment program of anal cancer has not been proposed to HIV infected MSM either so far.

In our study, we obtained a remarkable finding that HIV-positive MSM with higher CD4^+^ T cell level were more likely to be infected by HPV. However, by applying the Bruzzi method [38], it is estimated that avoiding having CD4^+^ T cell counts drop below 200 cells/μL 6–7 years prior to anal cancer diagnosis would prevent 20% of anal cancer cases, this estimate could rise to 49% or 79% by keeping CD4^+^ T cell counts higher than 350 cells/μL or 500 cells/μL, respectively. A cohort study from Swiss addressing the relationship between CD4^+^ T cell level and anal cancer risk suggested a rough estimate of the fraction of anal cancer [39]. The contradictory results compared with previous literatures urged us to further explore this finding. Otherwise, ART may not have been initiated early enough to have prevented the establishment of a large proportion of irreversible precancerous anal lesions [5,40,41]. If the risk of anal cancer could be largely attributable to HIV-related immunodeficiency as assessed by decreased CD4+T cell counts, early diagnosis of HIV infection and early starting ART might be key point to prevent anal cancer development among HIV infections [39–43].

Several limitations of this study should be kept in mind. Firstly, potential recall bias on sensitive questions could not be excluded completely because our questionnaires were interviewer-administered. Secondly, in terms of difficult sample acquirement and the potential limitation of enrollment methods, our study participants might not represent the general HIV-positive MSM in Xi’an City. Thirdly, due to the relative small sample size, the power of our study for association analysis is limited. Last but not least, cross-sectional study design has its limitation on association analysis. Therefore, our results need confirmation by further large-scale case-control studies or prospective studies.

In conclusion, high prevalence of anal HPV infection was observed among HIV-positive MSM in our investigation. Further studies are needed to estimate the risk of HPV related cancers and to explore strategies accordingly for intervention in this population. Developing targeted vaccination based on the most prevalent carcinogenic HPV types should be considered for different population in different areas.
